# Book Review

**Published:** 1936

**Authors:** 


### Reviews of Books

**Hero Dust.** By JAMES KEMBLE, Ch.M., F.R.C.S. Pp. xvi., 192. London: Methuen & Co. 1936. Price 6s.—In *Hero Dust* the author sets out to explain the lives and conduct of certain notable figures in history by a study of their diseases. His choice is varied, Mary Queen of Scots, Epicurus, Catherine the Great, Milton, Beau Brummell and Omar Khayyám. He has thought of them as patients, pondered on their ailments, wondered at their death and (in fancy) conducted their post-mortems. His explanation of the tragedy of Mary Queen of Scots is based on her pregnancies, one of which ended in the birth of an infant son, who (if he survived) became James I of England, and the other in her miscarriage soon after her marriage to Bothwell. Yet the known facts of Mary's tragic life give her an historical interest far more than any obstetrical speculations. If she had not been lovely and forlorn Scotland would never have made a national heroine of Mary. The life of Epicurus is perhaps the most interesting chapter in the book, because his writings and philosophy are presented very fully, the analysis is good, and the subject fascinating. His bladder trouble fortunately remains unmentioned until near the end, where it does no harm to the narrative. Catherine the Great is considered almost entirely from the view-point of her amatory excursions. But these are insignificant beside the success with which she ruled Russia. They do not explain her greatness, they scarcely serve to emphasize it. Here was a woman born of undistinguished parents, who cohabited monogamously with a series of twenty-three temporary husbands, who lived to the age of sixty-seven, earned the right to be reckoned among the greatest rulers, and won the affectionate admiration of the English letter-writer, Maria Wilmot. Surely she was something more than a mere nymphomaniac. There seems little excuse for Beau Brummell's presence as a patient in Mr. Kemble's consulting-room. True, he died with all the signs of sclerosis of the cerebral arteries, but this degeneration was the result rather than the cause of his mode of life. The sketch of Omar Khayyâm gives an enchanting picture of the poet's environment in Persia, but how much we know about the real Omar and how many of the quatrains attributed to him are his was a puzzle even to the late Professor E. G. Browne. In any case the absence of material for a psycho-pathological study makes the chapter on Omar the most charming in the book. John Milton we have left to the last because it is a longer study than the others, occupying 60 out of the 184 pages of text. The author seems to have little love for Milton. He dislikes his treatment of his wives and daughters, he dislikes his political pamphlets and dislikes most of all his views on divorce. He takes an unconscionable time in telling his readers that Milton suffered from "gutta serena" which, as it was accompanied by pain and haloes round the candle-lights, can only have been "glaucoma." Perhaps Mr. Kemble was not taught this as the classical illustration of glaucoma in his student days, as the reviewer was some forty years ago. The author declaims against the malignancy of Milton's political views, quotes Mark Pattison's description of Milton's language as that of the gutter and the fish market, and repeats Macaulay's advice to consign his prose essays to the dust and silence of the upper shelf. Yet there is something in Milton's prose besides malignancy and vicious invective. Like the Authorised Version of the Bible it remains living English and has, unknown to many writers, impressed its style on succeeding generations. The best American prose writers, whose forefathers crossed the Atlantic before the time of Dr. Johnson, are the truest heirs of Milton the prose-writer. Theirs is an enviable heritage. In spite of these contentions with Mr. Kemble's views his little book of essays has been irresistibly readable. After all, a dog presumably loves a bone even when he bites it most fiercely. He goes on growling and gnawing and enjoying himself just like a reviewer of *Hero Dust*.

**Johannes de Mirfeld of St. Bartholomew's, Smithfield: his Life and Works.** By Sir PERCIVAL HORTON-SMITH HARTLEY, C.V.O., M.A., M.D., F.R.C.P., and HAROLD RICHARD ALDRIDGE, M.A. Pp. xviii., 191. Illustrated. Cambridge: The University Press. 1936. Price 15s.—The writings of Johannes de Mirfeld are of very great interest as a *speculum* or mirror of contemporary thought and especially to those who are members of the old Bartholomew's foundation.

They are the first writings of a medical nature known to be associated with an English hospital, and perhaps for that reason a disproportionate value has been placed on John of Mirfeld's standing as a physician. He shows himself to be a kindly man and widely read, but his writings are entirely scholastic and contain no original matter. Mirfeld lived and wrote in the Priory in Smithfield towards the end of the fourteenth century; but he lived in the Priory as a "boarder." He was certainly not a medical man, nor was he a priest until near to the end of his life. There is a Chancery Inquisition Post-Mortem of the date 30th December, 1375, which states that John, the son of William de Mirfeld (*sic*) was at that time upwards of forty years of age. An extract from the Bishop of London's ordination lists, dated 19th September, 1394, describes John as "Acolyte," and adds "this man was not ordained" (*iste non fuit ordinatus*); on 6th March following he was advanced to be sub-deacon, and a year later to be deacon, and finally on 10th April, 1395, to be priest. This brings his age to close on sixty. He was not admitted to full membership of the Augustinian order, nor was he ever a canon. The executrix who proved his will described him as "Dominus" and "Capellanus," an unbeneficed priest who acted as a chaplain. No explanation is forthcoming of the reasons for his spending so many years of his life a resident in the Priory, educated like a cleric, but unordained and unprofessed. Here he devoted a great deal of time and industry to making compilations from the great authors of his own time and of antiquity. Greek, Arabian, Latin and Mediæval writers were all laid under contribution by him, yet over and over again he disclaims any part in the theories and opinions which he quotes so freely. His chapter on "The Signs of Death," even though the editors describe it as a "Mixture of Science, Nonsense and Superstition," and the chapter on "The Treatment of Consumption" are so well compiled and written that the reader cannot help wishing for more. Phthisis, defined as a wasting away of the whole body, arising from ulceration of the lungs, was a topic of special interest in Mirfeld's time owing to the illness of the popular hero, the Black Prince, whose death was ascribed to this cause. The editing of these writings has been done very thoroughly and with a wonderful charm. The choice of chapters for translation cannot be too highly praised. The editor's account of the practice of Medicine or Law by ecclesiastics is meticulously free from error. Research has identified Johannes de Mirfeld as the author of two volumes, *Breviarium Bartholomei* (which includes the *Sinonoma* found only in the MS. at Pembroke College, Oxford) and *Floriarium Bartholomei.* A simple reprint of the *Breviarium* alone would have made a volume of 2,400 pages similar to that now under review. Hence we feel that the editors deserve our best thanks for the scholarly selection they have managed to place at our disposal in so compact a book.

**The Cranial Muscles of Vertebrates.** By F. H. EDGEWORTH, M.A., M.D., D.Sc., Professor Emeritus of Medicine in the University of Bristol. Cambridge: The University Press. 1935. Price £5 5s.—Zoologists are indebted to Professor F. H. Edgeworth for this review of a specialist field in which he has been an important contributor for many years. *The Cranial Muscles of Vertebrates* collects together all the knowledge in this field, which in itself would be a signal service to comparative anatomy, but it does not stay here. This knowledge is arranged and analysed, and from the general principles collected together there emerges an attempt to follow the genetic relationships of the vertebrata and so ultimately "the phylogenetic history of man in the remote past." The work starts with a discussion of the mesoderm of the head and of the number of branchial segments in the primitive gnathostomatous vertebrate stock. The process of cephalization is followed, and shown to be essentially a backward extension of the cephalic mesoderm, resulting in the atrophy of the anterior body myotomes. This process is an important factor in the development of a neocranium out of the original palæocranium. From this starting-point the various muscle groups in the head are discussed one by one in great detail for each vertebrate order, and in addition there are important references to the innervation of the muscles and to the skeletal anatomy of the head where necessary. In the short space of a reviewer's note there is no room to indicate the amazing mass of facts which have been collected together and analysed. It must suffice to say that there is a thorough survey of every important problem in every group. Professor Edgeworth has not only read the literature of his field, he has in many cases examined the material on which other workers' conclusions are based, and he has personally made important contributions in every aspect of the field. The difficulties of his work were immense. Some of the vertebrates examined are very rare, and nothing but indefatigable interest could overcome the problems of merely obtaining them, let alone carrying out the examinations. At all points there is evidence of extraordinary care in the preparation and interpretation of sections and dissections. It is obvious that more than one specimen of each vertebrate has been examined, that sections have been cut in several planes, and that the data presented are objective facts and not subjective inference. Professor Edgeworth's final conclusions are by no means acceptable to all comparative anatomists. In the last two chapters is a summing up of his theories. A discussion of the homologies and innervation of the cranial muscles shows how difficult it is to obtain reliable criteria of homology, which are essential for making phylogenetic analyses. The factors which can confuse the issue are tabulated, discussed and illustrated, and one can only feel that a writer able to assess these factors so clearly cannot fail to be a reliable guide. With this feeling one becomes more assured in turning to the final discussion of the ancestral form and history of the cranial muscles. The facts are well weighed, and the logical conclusion is reached that the mammals have arisen from an amphibian stock, and not a reptilian, as the palæontologists in particular believe. The palæontological viewpoint rests essentially upon a change in the articulation of the jaw, which has been sought in the reptiles. Edgeworth's detailed evidence, however, strongly suggests that the change from an incudo-meckelian to a squamoso-mandibular jaw-joint occurs *within* the mammals, which is a point of major importance. In addition there is a battery of other cranial features (particularly regarding the masticatory muscles, the levator hyoidei, the laryngei and the origin of the six external ocular muscles from the pre-mandibular somite) which suggests a direct descent of the mammals from primitive amphibia. Professor Edgeworth sets out to solve a phylogenetic problem, but all the time his evidence recalls another field, the experimental study of embryological development. His problem lies in evolutionary time, but he adduces evidence and ideas which will be of value in the study of individual development. His work is an immensely valuable contribution to the central problem of biology—evolution. *The Cranial Muscles of the Vertebrates* is of the greatest importance to any study of zoology, because of the width of its contacts. It reaches beyond the purely descriptive. This is in large part due to the concise and logical presentation of the data, to the flair which the writer possesses for making analyses and syntheses of multitudinous facts in a few words, and to the excellence of the mechanical aspects of production. There is an index of cranial muscles, cartilages, and nerves, and a complete list of terms and abbreviations which are always so well chosen that in looking at a figure one hardly has to refer to the index. The table of synonyms is stated by the author to be far from complete. This may be so, but it extends through forty pages and does much to clear up the difficulties which arise from the lack of uniform nomenclature. A long list of references collects together all of importance to a date just prior to publication. And finally there are the figures, 841 in number, occupying nearly 200 pages. The majority are original, but derived figures are also used so that every point is well and clearly illustrated with excellent drawings. The Cambridge University Press must be congratulated on the style and production of the book. This work is essential to any specialist worker in this field, and cannot be neglected by other zoologists or anatomists simply because of its very wide implications. It is a vast storehouse of material so well arranged and presented that at every moment it serves not only as a store but as a stimulus for new ideas and for new lines of work. It is indeed a commendable pattern of logic and objective study.

**Baillière's Synthetic Anatomy.** 14 parts. By J. E. CHEESMAN, L.M.S.S.A., D.P.H., R.C.P.S. Illustrated. London: Baillière, Tindall & Cox. 1936. Price 3s. per part. —This is certainly the most ingenious and original atlas of human anatomy; but it is also by far the most complicated and difficult to understand, and we cannot see that it attains its "synthetic" object. In the sections on arm and leg certainly the superimpositions of the transparent drawings begin by being clear and informative, but naturally the deeper the dissection the more structures are revealed, and clarity is lost. Indeed, the confusing mass of detail presented in the pterygo-maxillary region renders the interpretation and relation of structures impossible; especially since here, as indeed throughout, the depth or plane of dissection is not specified, nor is there given any indication of what each drawing is particularly designed to show. The section of the brain is the most abstruse and indefinite of all, and we fail to see its value; there is no view of the convolutions, the base, or mesial section, but merely a series of diffuse, detailed and disconnected sketches which convey the impression that the brain is a pancake here cut into thin slices. And though we are aware that the contents of the orbit are diverse and complex in arrangement, we have not hitherto been confronted by them in the form of a pyrotechnic nightmare. The junior student studying anatomy for the first time with this atlas may well be appalled and discouraged by the intricacies and details of the subject; the senior, using it for revision, will surely find his recollection of actual dissections disturbed, distorted, and even devastated. Both, we assert, would gain far more advantage in expenditure of time by reading their text-book and looking at its contoured figures (which do remind one of dissections) than by poring over these flat and flattening complexities until exhausted to the point of exasperation. The most that either could say is that his wondering pencil had checked off piecemeal every single structure in the body by wading laboriously through the bald list of names in the map references, with little connection between them and, of course, no thought of their functions. He will likely end with a befogged impression of any region of the body—and a headache. Could he obtain from even a prolonged study of these fluctuating leaves a clear idea of, for instance, such a simple thing as the origin, course and distribution of the median nerve? We doubt it. The technical production of this work of great labour is a fine tribute to the publishers, printers and binders, and this at least justifies the price of 45s. The student or practitioner may be bemused by its novelty, and if he can afford it buy it, feeling he likes to have it. It will lie on his shelves. It is pertinent to add that any well-illustrated textbook of developmental, descriptive, applied and surface anatomy will cost him less and be of far greater practical value. He will need to have it, and will keep it on his desk.

**Vitality and Energy in Relation to the Constitution.** By T. E. HAMMOND, F.R.C.S. Pp. xii., 314. Illustrated. London: H. K. Lewis & Co. 1936. Price 12s. 6d.—This sagacious book is complementary to Mr. Hammond's former publication. Enquiring why so many fail of vigorous life he discusses two types, the hyposthenic and the hypersthenic, each with its assets and liabilities: their reaction to bacterial invasion; the nervous system in the control and defence of the body, and as holding in its recesses the emotional centres of faith or fear. Life moulds its forms, adapts itself to change, answers to habit. The author is discursive and admits some repetition ; literary form, perhaps, would gain by correction and revision. Clinical illustration is drawn from old masters, from the surgical life, from battle fronts and men massed there, and from what one learns from one's own grief and pain. Man is a sensitive, delicate creature, ill-fitted to his cosmos. For the author there is a glamour in the daily practice of the village doctor : his is a lore which helps his neighbours, for he knows their idiosyncracies and nuances.

**Symptoms and Signs in Clinical Medicine.** By E. NOBLE CHAMBERLAIN, M.D., M.Sc., M.R.C.P. Pp. xii., 424. Illustrated. Bristol: John Wright & Sons. 1936. Price 25s.—This is an excellent publication, interestingly written, profusely illustrated with illustrations which really do elucidate the text, and most superbly produced. The aim of the book is to present to the student (and which of us is not still a student?) the technique of physical examination and of eliciting physical signs. Then, in the words of the author, "an attempt has been made to take the student a stage further to the visualization of symptoms and signs as forming a clinical picture of some pathological process." In this endeavour he has admirably succeeded. After chapters upon the routine of interrogation and examination, and upon the external characteristics of disease he proceeds to take the various systems of the body and to go through them, pointing out how "symptoms and signs are pieced together in the jigsaw puzzle of diagnosis." Excellent chapters upon the examination of sick children, the simpler medical operations and instrumental investigations, and then one upon the ordinary examinations of clinical pathology and biochemistry follow the main part of the book. As is essential in a work of this character, the author is dogmatic in his statements and clear cut in his views. The work is really a glorified *Clinical Methods*, combined with a simple index of diagnosis, and the predominant feeling in at least one reader's mind is one of regret that if such works were produced in his student days he never had the good fortune to come across one. But surely one of the simpler diagrams of the false and true images in diplopia would have been easier to understand. The author is to be warmly congratulated upon this splendid production, and, as he himself is the first to acknowledge, he owes a great deal to his publishers for their excellent work.

**The Early Diagnosis of Malignant Disease.** By MALCOLM DONALDSON, F.R.C.S., STANFORD CADE, F.R.C.S., WILLIAM DOUGLAS HARMER, M.C., F.R.C.S., R. O. WARD, M.Ch., F.R.C.S., and ARTHUR TUDOR EDWARDS, M.D., M.Ch., F.R.C.S. Pp. viii., 168. London: Oxford University Press. 1936. Price 8s. 6d.—In this small volume five authors have collaborated in presenting to the general practitioner the essential points in making a provisional diagnosis of cancer, and in urging the necessity of sending the patient to some hospital or other institution for expert opinion at the first suspicion of malignancy. The twelve chapters cover practically the whole range of the commoner malignant growths with the notable, and regrettable, exception of intracranial neoplasms. In most cases the admirable procedure is followed of contrasting forcibly the *early* symptoms and signs with the generally accepted classical evidences of the disease. The great value, and the limitations, of modern aids to diagnosis are stressed, in particular the expert use and interpretation of radiological, endoscopic and pathological examinations. In connection with the last-mentioned it is interesting to note that the dangers of biopsy of malignant tumours for exact histological diagnosis are dismissed as unfounded, except in the case of bone sarcomata. The "precancerous" conditions receive due attention, and treatment is indicated only in so far as its results emphasize the enormous value of early diagnosis and appropriate therapy by experts. The book is well indexed and, probably wisely, devoid of any illustrations or reference to current or past literature. Though in places the brief pathological descriptions are not strictly accurate, the information is fully up to date, and the volume should prove invaluable to senior students and young practitioners, and a welcome encouragement to all whose aim it is to reduce the toll of death and suffering from cancer.

**An Index of Treatment.** Edited by ROBERT HUTCHISON, M.D., LL.D., F.R.C.P. 11th Edition. Pp. xvi., 1,020. Illustrated. Bristol: John Wright & Sons. 1936. Price 42s.—It is just over four years since the last revision of this standard work on treatment was published, and both the editor and the publishers are to be congratulated on their determination to keep the *Index of Treatment* constantly up to date. Many of the articles have been completely re-written and many new articles on subjects not dealt with in previous editions are included. Among the new articles are to be found the treatment of such important conditions as agranulocytosis, allergic diseases, alkalosis, functional diseases of the gall-bladder and the anæmias of childhood. The last-named (anæmias of childhood) form a most important and interesting group, which is fully and admirably dealt with. One feels that it is unnecessary to recommend the book; it is too well known. All that seems to be called for is to draw attention to the appearance of a new edition. Every doctor knows he has to have it.

**Bacterial Endocarditis.** By C. BRUCE PERRY, M.D., M.R.C.P. Pp. viii., 137. Illustrated. Bristol: John Wright & Sons. 1936. Price 10s. 6d.—We welcome this book describing the work carried out by Professor Perry, under the auspices of the R. L. St. John Harmsworth Memorial Research Fund. The object of the Fund is to promote the advancement of the investigation of bacillary or infective endocarditis. This is the first volume issued by the trustees of the Fund, and it is hoped that further work on the subject of endocarditis will be published in subsequent volumes. The book covers a large field in a small space. The clinical account of bacterial endocarditis is admirable and covers the ground admirably. The illustrations, which are in black and white only, represent the pathology of the condition excellently. One feels, however, that the illustrations are so good that it is a pity several of them were not reproduced in colour. The hæmatology and bacteriology is particularly well surveyed. In an appendix by Dr. D. M. Lloyd Jones a full and interesting account is given of an experimental study of bacterial endocarditis carried out in the Pathological Department of St. Bartholomew's Hospital under a grant from the Harmsworth Memorial Research Fund. This volume, which is published by John Wright & Sons at a most reasonable price, will prove of the greatest value to all practitioners as well as research workers.

**The Treatment of Asthma.** By F. T. HARRINGTON, M.R.C.S. Pp. x., 112. Illustrated. London: H. K. Lewis & Co. 1936. Price 6s.—The author maintains that in the treatment of asthma every system and function of the body has to be considered. As to diet, compatibility is of great importance—that is, either proteins or carbohydrates may be taken at the same meal as fruit and vegetables, but the first two together are incompatible: further, all meals should be taken dry, and what drink is needed between meals, avoiding milk. Constipation is universal: the patient must go to stool immediately after *every* meal. Breathing exercises are an essential part of every treatment, as the asthmatic does not employ his diaphragm—the author asserts that at the end of expiration muscular effort of the relaxed diaphragm forces it *up*. Clothing, baths, packs, Turkish baths and exercise (walking and skipping) are also prescribed. Finally, the condition of the nose demands attention: the most common abnormality is ethmoiditis (apparently that variety with no clinical, radiological or bacteriological signs), and this is treated as usual by means of the "ethmoid pack": another "desensitizing" treatment is "diastolization," or massage of the nasal passages with inflatable rubber bougies, a method the author considers far superior to cauterization. The remainder of the book is devoted to case reports, diet charts and recipes. It is difficult to believe that asthma can be as uncomfortable as the treatment. The book is well produced, though spelling solecisms (*e.g.* "mucus membrane") are irritating.

**Foundations of Short Wave Therapy: Physics—Technics—Indications.** By WOLFGANG HOLZER, Dr.Ing., and EUGEN WEISSENBERG, Dr.Med. Pp. 228. Illustrated. London: Hutchinson. 1935. Price 12s. 6d.—It is necessary when publishing a book dealing with a new form of ray therapy to give an adequate description of the underlying physical conceptions, but in this book the authors have devoted more than half its volume to such advanced physics that it is necessary to be a highly-trained physicist in order to understand it; and the physician and the biologist, for whom they say this book is primarily intended, would, therefore, find a great part of it unintelligible. A short, more elementary physical introduction dealing with the details of the apparatus and its method of use would render the book more valuable, since the beginner in this branch of therapy is given relatively little information of this nature. The part dealing with the medical uses is clear and interestingly written as far as it goes, and one is given the impression that the writers have a great deal more information which they do not feel is sufficiently proved to justify its publication. The book is clearly printed, has an excellent bibliography, but a rather small index. The translators are to be congratulated on their work.

**Emergency Surgery.** By HAMILTON BAILEY, F.R.C.S. Second Edition. Pp. xii., 842. Illustrated. Bristol: John Wright & Sons. 1936. Price 50s.—The appearance of a second edition bears witness to this book being a boon to surgical students, qualified and qualifying. An added appeal in the new edition is that the whole work is bound in one cover instead of two volumes. Doctors are deluged with literary outpourings, and readily appreciate every simplification and compression of books as of other essential paraphernalia. By restricting himself to what is of wholly practical value, the author encompasses the whole range of surgical emergencies completely. He is concise, but not to the exclusion of precision. Due regard is paid to the choice of instruments and minutiæ of technique, on the faithful performance of which successful operating so often depends. Brightening up the sterner descriptions on surgical procedure are beautiful illustrations, many of which are coloured. Thrilling stories of actual cases, rescued by the author from grievous perils, emphasize the value of the methods he advocates, as, for instance, that told of the boy with injury of the lung (p. 432, *et seq.*). Occasional epigrams sprinkle the text and serve acceptably to "point a moral or adorn a tale." These fit aptly with the vigorous and impressive style of the production. Among the subject matter, blood transfusion receives the merit it deserves in the opening chapter. Due attention is given to another popular helpmate of modern surgery, spinal anæsthesia. The bulk of the book deals most commendably and in fair proportion with the surgical crises of trunk, head and limbs, and in many parts improves on its predecessor. Thus, retrograde jejuno-gastric intussusception is now included, as befits it, among the complications of gastro-enterostomy. The more radical measures for treatment of ischiorectal abscess is a commendable addition. There is also told and depicted the Cabot method of nephrostomy. Where masses of detailed facts and reference have to be marshalled, a few errata are but human. Thus, the meaning of the arrow in Fig. 208, p. 223, is obscure, while, presumably, the inversion of the illustration on intercostal anæsthesia (Fig. 411, p. 422) and of the tympanic membrane (p. 743), are oversights. Lane's forceps (Fig. 171, p. 185) are surely too coarse for handling bowel. Is the picture of an ampoule of serum (Fig. 175, p. 194) worthy of space in this concise compendium? However, if the readers of this book fail to do justice to their patients' needs, they must blame themselves and not it.

**The Early Diagnosis of the Acute Abdomen.** By ZACHARY COPE, M.D., M.S., F.R.C.S. Seventh Edition. Pp. xiv., 254. Illustrated. London: Oxford University Press. 1935. Price 10s. 6d.—This is an excellent and well known monograph and has now reached its seventh edition. Several small alterations have been made, and some interesting and instructive observations on the value of X-rays in the diagnosis of intestinal obstructive conditions have been added. As its name indicates, the book confines itself entirely to the question of diagnosis, with a wealth of detail in regard to symptoms and signs. The first chapter deals with several sound elementary principles and observations in relation to diagnosis, and in the second chapter there is an admirable specimen form for history-taking in acute abdominal cases. The book is well printed in good size type, and there are numerous good diagrams. There is an accurate and detailed index which makes reference to any particular point quite easy. This is a valuable guide to practitioners in this particularly difficult and urgent type of case.

**Diseases of the Nose, Throat and Ear.** Edited by A. LOGAN TURNER, M.D., LL.D., F.R.C.S. Fourth Edition. Pp. xx. 473. Illustrated. Bristol: John Wright & Sons. 1936. Price 20s.—The appearance of another edition of this excellent work shows that it continues to occupy the place it so well merits as the leading text-book in diseases of these parts. The present volume closely follows the lines of previous issues. New chapters have been added on nasal polypi and on allergy, and the section on brain abscess has been amplified. The illustrations are numerous, well chosen and excellently reproduced, the printing and general arrangement are all that could be desired, there is a full and complete index, and, in short, both authors and publishers have fully maintained the standard of really first class work which they have set themselves.

**Aural Therapy in Relation to Deafness.** By D. F. FRASER-HARRIS, M.D., D.Sc., F.R.S.E. Second Edition. Pp. ii., 45. Illustrated. London: Sterling Medical Publishing Co. 1936. Price 7s. 6d.—The most astonishing thing about this short monograph is that it has reached a second edition. It is apparently addressed to the deaf layman, with a view of acquainting him with recent progress in audiometry and the prescription of aids to hearing specially modified for the needs of each patient: would that practice were as simple as the theory! The statements that hydrogen peroxide is a simple solvent of cerumen; that otosclerosis is a rheumatic condition; and "since we know that too much smoking causes irregular action of the heart, hyperacidity and blurred vision, it is impossible that the nerve of hearing should escape the poisoning," sufficiently indicate the scientific value of the pamphlet, and such spellings as "asperin" its literary standard.

**The Nature of Disease Journal. Vol. III.: The Common Cold and Influenza.** By J. E. R. McDONAGH, F.R.C.S. Pp. vi., 152. London: William Heinemann & Sons. 1936. Price 12s. 6d.

"A fool is bent upon a twig, but wise men dread a bandit."
Which I think must have been clever, for I didn't understand it.

The pathetic reaction of Ferdinando to Mister Martin Tupper's cryptic utterance will occur to many of those into whose hands this volume comes. Only the favoured few who have already mastered the author's vocabulary and preliminary theses can appreciate its full beauty and value. It is devoted to the epidemiology of the common cold and influenza, with special reference to the mutation of bacterial species under the influence of cosmic rays! It would appear that the astrology of ancient Nineveh was in reality an exact science. But we must remember that our author considers "disease" as practically synonymous with "biology." Both the large size of pre-historic Saurians, and the cerebral development of *homo sapiens* are aspects of DISEASE. Even the uninitiated, however, cannot fail to observe that treatment has become very much simplified during the course of ten years: for example, "The patient abstained from butchers' meat, eggs and milk, had a course of colonic lavage, took ichthyol and thyroid internally and received injections of Solganol B., of his fæcal vaccine, and of streptococcal anepidem" (and recovered!).

**A Text-Book of Psychiatry.** By D. K. HENDERSON, M.D., F.R.F.P.S., F.R.C.P., and R. D. GILLESPIE, M.D., F.R.C.P. Fourth Edition. Pp. xii., 602. London: Oxford University Press. 1936. Price 18s.—This book covers the scope indicated by the title, but, in addition, includes subjects not usually classified under the term psychiatry. In this country psychiatry usually excludes mental deficiency and the psychoneuroses, the latter being included under medical psychology. The value of the book is enhanced by these inclusions. The book is written in as simple language as possible, as it is intended for students and general practitioners, but much information would be of little interest to the latter class in this country. For instance, statistical information with regard to the psychoses in the United States without figures for this country is of little value, particularly as some of the figures have not been brought up to date and relate to conditions existing seventeen years ago. Details of sample cases are well described, the patients appearing as living personalities. In the section on mental defect definite mental age limits are given for the idiots and the imbeciles. The fixing of such limits has now been generally given up, as cases occurring outside the arbitrary limits of up to two years for idiots and from two to seven years for imbeciles must sometimes be classified in the other group. The type is clear, the index full, and a short bibliography is appended. The popularity of this volume is shown by the appearance of four editions in nine years. This popularity is well deserved, and the book can be recommended to all those beginning a study of psychiatry.

**Warwick and Tunstall's "First Aid" to the Injured and Sick.** Fifteenth Edition. Edited by F. C. NICHOLS, M.C., M.B., Ch.B. Pp. x., 298. Illustrated. Bristol: John Wright & Sons Ltd. 1936. Price 2s. 6d. net.—Warwick and Tunstall is the standard book for all first-aid training, and it is satisfactory to see that the publishers and editor are determined that it shall be kept up to date. The fifteenth edition, like the thirteenth and fourteenth, has been edited by our colleague, Mr. F. C. Nichols, who has done his work admirably. The general plan of the book and its illustrations remain unchanged and as good as ever. The chief addition is a chapter on "Gas Poisoning in Warfare," a subject of which the editor has abundant first-hand knowledge, although he modestly says that the chapter is based on the *Official Air Raid Precautions Handbook No. 2.* We hope that the information contained in this chapter will reach a wide public. The chief value of poisonous gases in warfare is their power of causing panic; as the author correctly observes, even in the case of mustard gas, "recovery is usual." Precautions against gas are easy to arrange and most effective. Panic is the fighting factor, and a public that has read this chapter will not permit itself to be thrown into a panic. It is interesting to see air transport of casualties taking its place as an ordinary method. Without wishing to be unduly critical, one wonders whether the treatment of carbon-monoxide poisoning ought not to find a place in future editions. Mine-rescue is, of course, highly specialized work, but the employment of oxygen inhalation apparatus, oxygen-tents and Drinker's respirator is rapidly approaching the point where they should be included in "First Aid to the Injured."

**Vitamins and Other Dietary Essentials.** By W. R. AYKROYD, M.D. Second Edition. Pp. xii., 226. London: William Heinemann Ltd. 1936. Price 7s. 6d.—A second edition of Aykroyd on Vitamins will be warmly welcomed. In his first edition the author produced a book which was acceptable to the medical and general reader alike. The new edition is no less readable, and it is a matter for congratulation that the temptation to include specimen dietaries and tables of food values has been resisted. In a scientific discussion of fundamentals these would be out of place. The author presents scientific facts in a form and in a literary style that makes them intelligible to a reader with no physiological knowledge. Yet at the same time the established facts are so complete and so well arranged that there are few books to which the practitioner can better refer for the information he needs in his every-day practice. The habit of writing decimal parts without a cypher is dangerous, as the figures may easily be misread; thus, on page 193 are to be found: " ·62 of a kilogram " and " ·4 of a centimetre." The correct printing of those figures is 0·62 and 0·4. Dr. Aykroyd has recently (in 1935) taken on the duties of Director of Nutrition Research under the Indian Research Fund Association, and in this edition a great deal of interesting information has been added on the enormously varied dietary problems of the Indian peoples; for India remains one of the most dreadful fields for the study of food deficiencies of every sort. Books like Aykroyd's make one feel little doubt that economic and dietetic problems are of greater moment in world politics to-day than the dynastic and religious difficulties which used to form the basis of school history books.

